# CarboCell G/C provides sustained high local bone antibiotic levels with minimal systemic exposure, supporting its therapeutic potential in orthopedic infection management

**DOI:** 10.5194/jbji-11-257-2026

**Published:** 2026-05-08

**Authors:** Nicole Lind Henriksen, Andrea René Jørgensen, Michal Poborsky, Christoph Crocoll, Niranjan G. Kotla, Catrine Jyde Berthelsen, Mats Bue, Louise Kruse Jensen, Anders Elias Hansen, Jonas Rosager Henriksen

**Affiliations:** 1 Department of Veterinary and Animal Sciences, University of Copenhagen, Copenhagen, Denmark; 2 Department of Orthopedic Surgery, Aarhus University, Aarhus, Denmark; 3 Department of Clinical Medicine, Aarhus University, Aarhus N, Denmark; 4 Department of Plant and Environmental Sciences, University of Copenhagen, Denmark; 5 Department of Health Technology, Technical University of Denmark, Kgs. Lyngby, Denmark

## Abstract

**Introduction**: Achieving sustained local antibiotic concentrations is critical for managing orthopedic infections. This proof-of-concept study evaluated release kinetics, local/systemic concentrations, and histological responses to CarboCell G/C, an injectable gentamicin- and clindamycin-eluting depot in a porcine bone void model. **Methods**: CarboCell G/C containing 111 mg g^−1^ gentamicin-docusate and 70 mg g^−1^ clindamycin was injected into and around a tibial bone void in 11 pigs. Antibiotic concentrations in target tissues and systemic spillover were measured at 1–12 h by microdialysis and liquid chromatography coupled to tandem mass spectrometry (LC-MS/MS) and in tissue homogenates at 1, 3, and 7 d. Release was quantified by high-performance liquid chromatography (HPLC), and tolerability was assessed histologically. **Results**: Microdialysis showed high stable antibiotic tissue levels during the first 12 h with minimal systemic spillover. Peak gentamicin and clindamycin concentrations in bone were 730 ± 170 and 3700 ± 700 µg g^−1^ and 42 ± 15 and 360 ± 120 µg g^−1^ in soft tissue, respectively. Tissue half-lives ranged from 43–62 h for clindamycin and 40–46 h for gentamicin. Recovered depots had released ∼ 30 %–50 % of their antibiotic content at day 1 and ∼ 60 %–80 % by day 7. Depots elicited a mild–moderate local inflammatory response. **Conclusion**: CarboCell G/C provides sustained high local antibiotics for at least 7 d with minimal systemic spillover, supporting further evaluation in orthopedic infection management.

## 1 Introduction

Orthopedic infections are complex and challenging to treat for several reasons, two of which are the presence of bacterial biofilms and dormant persister cells (Zimmerli et al., 2012). To address these challenges, local antibiotic delivery technologies have gained increasing interest (Jennings et al., 2026). Compared to systemic antibiotic therapy, these technologies offer several potential advantages, including (i) achieving high concentrations of antibiotics in infected regions, (ii) minimizing the risk of antimicrobial resistance, (iii) reducing systemic spillover and associated adverse effects, and (iv) enabling the incorporation of tissue-regenerative biomaterials (Steadman et al., 2023; Jennings et al., 2026). Several antibiotic-eluting biomaterials are available, including collagen, polymethylmethacrylate (PMMA), hydroxyapatite, and calcium sulfate, where cement and void filler technologies are the most commonly described (Steadman et al., 2023). However, concerns about their release kinetics have been raised. For example, for antibiotic-eluting PMMA bone cement, a consensus statement by experts in musculoskeletal infection highlights the risk of early release followed by prolonged subtherapeutic levels of antibiotics (Schwarz et al., 2021). In a systematic review, the total in vitro release of different antibiotics from PMMA bone cement has been reported to be below 10 % even after up to 8 weeks (Martínez-Moreno et al., 2017). In contrast, hydroxyapatite and calcium sulfate biomaterials demonstrate rapid early release of antibiotics (total release > 95 % within 24 h in vitro) (Bezstarosti et al., 2024). Antibiotic delivery technologies with improved and fully characterized release kinetics are therefore warranted. Additionally, data describing antibiotic tissue penetration from such technologies are limited, often restricted to measurements of antibiotic release from explanted biomaterials (Minelli et al., 2004) or measurements of antibiotic concentrations in wound exudate (Fleiter et al., 2014; Colding-Rasmussen et al., 2018). However, such measurements may not necessarily reflect target tissue concentrations, which are central to therapeutic activity (Fink et al., 2011; Steadman et al., 2023). Few studies have reported on concentrations of antibiotics in tissue (Fink et al., 2011; Klinder et al., 2019). Generating data on antibiotic tissue distribution after local delivery is essential to determine whether therapeutic concentrations are achieved and maintained at the target site and guide their clinical use (Müller et al., 2004).

Building on our prior results demonstrating that CarboCell G/C, a dual gentamicin–clindamycin antibiotic-eluting depot technology, can successfully eradicate implant-associated osteomyelitis in a porcine model without systemic antibiotics (Henriksen et al., 2024), this proof-of-concept study aims to characterize the pharmacokinetics and local tolerability. Specifically, we investigated local and systemic concentrations of gentamicin and clindamycin following administration of CarboCell G/C into a bone void.

## 2 Materials and methods

### 2.1 CarboCell G/C formulation

CarboCell was formulated as previously described (Henriksen et al., 2024). A total of 111 mg gentamicin-docusate and 70 mg desalted clindamycin were dissolved per gram CarboCell. In this study, 2.5 mL of CarboCell G/C, comprising 42 mg gentamicin and 158 mg clindamycin, was injected into each leg of the pigs.

### 2.2 Animal studies

Given the exploratory pharmacokinetic nature of the study, sample size was determined based on feasibility and consistency with previous large-animal pharmacokinetic investigations.

#### 2.2.1 Microdialysis (1–12 h)

Two 2- to 3-month-old Landrace female pigs weighing 34 and 35 kg were sedated with tiletamine–zolazepam (Virbac, France), ketamine (MSD Animal Health, USA), butorphanol (Orion Pharma, Finland), and xylazine (ScanVet Animal Health, Denmark) and anesthetized with propofol (B.Braun, Germany) and fentanyl (Fresenium Kabi, Germany). A hole was drilled into both proximal tibial bones and expanded with a bone curette to create bone voids of ∼ 1 cm^3^. CarboCell G/C was injected into the cavity (1 mL), surrounding trabecular bone (1 mL), and soft tissue (0.5 mL), and the wound was closed in three layers (periosteum, soft tissue, skin). A type 70 microdialysis catheter with a 20 mm membrane and 20 kDa cut-off (M Dialysis AB, Stockholm, Sweden, and 107 microdialysis pump (M Dialysis AB), perfused with 0.9 % NaCl at a flow rate of 1 µL min^−1^) was placed in the bone void–soft tissue interface. The method has been described in detail (Jørgensen et al., 2024). CarboCell G/C injection was time zero. Dialysates were collected initially every 30 min and every 60 min from after 2 h. Blood samples were drawn every hour from 60–720 min, and plasma was collected (3000 × g , 10 min, 5 °C). Catheters were calibrated by retrodialysis (1 mg mL^−1^ clindamycin and 1 mg mL^−1^ gentamicin). Pigs were euthanized using pentobarbital (Scanvet). Plasma and dialysates were stored ( - 80 °C) until liquid chromatography coupled to tandem mass spectrometry (LC-MS/MS) analysis.

#### 2.2.2 Tissue samples (1, 3, and 7 d)

**Figure 1:**
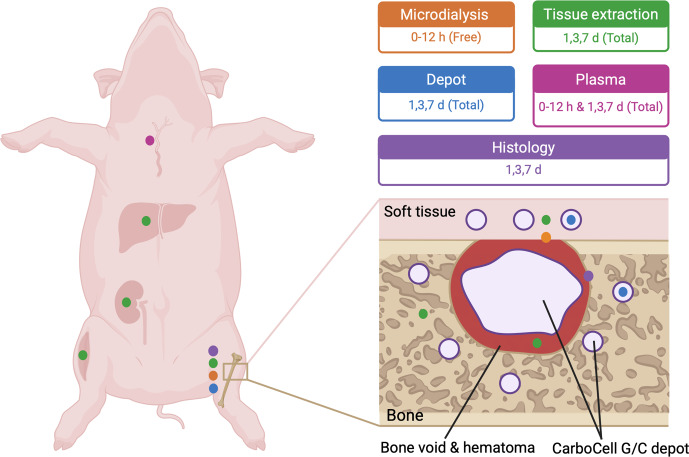
Pig study design.

Nine 2- to 3-month-old SPF Landrace female pigs (30–35 kg) were subjected to the protocol as described above; however, the procedure was only conducted in the right tibia. Animals received perioperative analgesia (meloxicam PO, Boehringer–Ingelheim) up to 7 d and were monitored in accordance with institutional animal welfare guidelines. Pigs were randomly selected, sedated, and euthanized after 1, 3, or 7 d ( n= 3 per timepoint). Blood was drawn prior to surgery and euthanasia, after which it was centrifuged, and plasma was collected. During necropsy, the surgical wound was opened, and soft tissue samples above the bone void were collected. The tibial bone was sagittally sectioned through the void. From one half, samples of the void hematoma and bone tissue below the bone void surface were collected. The other half was placed in 10 % neutral buffered formalin. Kidney, muscle, and liver tissue samples were also collected. Samples were stored ( - 80 °C) until LC-MS/MS analysis. CarboCell depots were collected for high-performance liquid chromatography (HPLC) analysis of antibiotic release (Fig. 1).

#### 2.2.3 Antibiotic release in mice

Antibiotic release over 21 d in mice was included as a secondary endpoint and performed as previously described (Henriksen et al., 2024). Briefly, two CarboCell G/C depots (50 µL ) were injected subcutaneously into the back of 15 anesthetized female, 8- to 9-week-old Balb/C mice (Taconic). After 1, 3, 7, 14, and 21 d, three mice were euthanized by cervical dislocation, and depots were collected.

### 2.3 LC-MS/MS

#### 2.3.1 Sample preparation

Samples were prepared as previously described (Mikkelsen et al., 2025). Frozen tissue samples were ground in liquid nitrogen. Weighed tissue sample (50–100 mg) were placed in 2 mL low-bind reaction tubes, and extraction was done in three steps. The first extraction was done by adding 500 µL 50 % acetonitrile in Milli-Q water ( v/v ) containing 0.1 % formic acid ( v/v ) and 500 ng mL^−1^ leucine–enkephaline as internal standards; the mixture was then vortexed and incubated for 20 min in a shaker at 25 °C at 1000 rpm. Samples were vortexed and centrifuged for 10 min at 10 °C and 21 000 ×g . The supernatant was transferred to a new tube. The second extraction was performed with 500 µL 50 % methanol in Milli-Q water ( v/v ) containing 0.1 % formic acid ( v/v ) and 500 ng mL^−1^ leucine–enkephaline as internal standards, and the third was performed with the same solvent as for the first extraction. Both were followed by the same steps for vortexing, shaking, and centrifugation. Extracts were pooled and diluted 20 × with 0.1 % formic acid in water ( v/v ) and filtered through 0.22 µm filter plates prior to LC-MS/MS.

#### 2.3.2 LC-MS/MS

LC-MS/MS was performed as previously described (Mikkelsen et al., 2025). The limit of quantification (LOQ) was 1 ng mL^−1^ for gentamicin and 0.6 ng mL^−1^ for clindamycin. Leucine–enkephalin was used as an internal standard for injection control and correction of inter-injection variation. The nominal LOQ for gentamicin and clindamycin in tissues was 0.6 and 0.4 µg g^−1^, respectively, whereas microdialysis and plasma samples had a nominal LOQ of 0.01–0.02 µg mL^−1^ for gentamicin and 0.006–0.01 µg mL^−1^ for clindamycin. Values below the LOQ were not included.

### 2.4 HPLC

CarboCell G/C depots were analyzed by HPLC, as previously described with the following modifications (Henriksen et al., 2024). The solvent system consisted of mobile phase A (5 % MeCN, 20 mM HFBA in water) and mobile phase B (20 mM HFBA in MeCN) with a gradient of 0 % B for 3 min, 0 %–50 % B over 4 min, 50 %–65 % B for 9 min, 65 %–100 % B for 30 s, 100 % B for 2 min, 100 %–0 % B for 30 s, and 0 % B for 4.5 min. Standards of 1000, 500, 250, 125, 62.5, and 31.25 µg mL^−1^ gentamicin and clindamycin and 10, 5, 2.5, 1.25, and 0.625 mg mL^−1^ SuBen were included.

### 2.5 Histology

Tissue sections (4–5 µm ) of formalin-fixed, decalcified, and paraffin-embedded bone were stained with H&E and Masson's Trichrome. Histological evaluation was performed using predefined semi-quantitative criteria. The evaluating pathologist was aware of the experimental condition but blinded to the sampling time point. Within the bone area with surgery-related changes, defined as the distance from the bone void border to the normal trabecular bone and marrow, the morphological tissue response to CarboCell G/C was described, including categorization of inflammatory response (mild, moderate, or severe). Furthermore, fibroplasia (percentage of collagen in 10 × 20 fields translated to a scale of 0–5) and osteoclast counts (maximum 10 cells counted in 10 HPFs) were scored as previously described (Henriksen et al., 2024).

### 2.6 Data analysis

Pharmacokinetic data were generated in GraphPad Prism version 10 (GraphPad Software, USA). Pharmacokinetic and release data are presented as means ± SEM, and histology data are presented as means ± SD.

Bone void was created in the proximal tibia, followed by injection of CarboCell G/C into the bone void and adjacent bone and soft tissue. Antibiotic concentrations were measured at the bone–soft tissue interface (orange); in bone, soft tissue, liver, muscle, kidney, and void hematoma at days 1, 3, and 7 (green); and in plasma (pink). Antibiotic release from depots recovered from bone and soft tissue was assessed at days 1, 3, and 7 (blue). Bone samples were collected at days 1, 3, and 7 for histological evaluation (purple).

## 3 Results

### 3.1 Tissue antibiotic measurements

**Figure 2:**
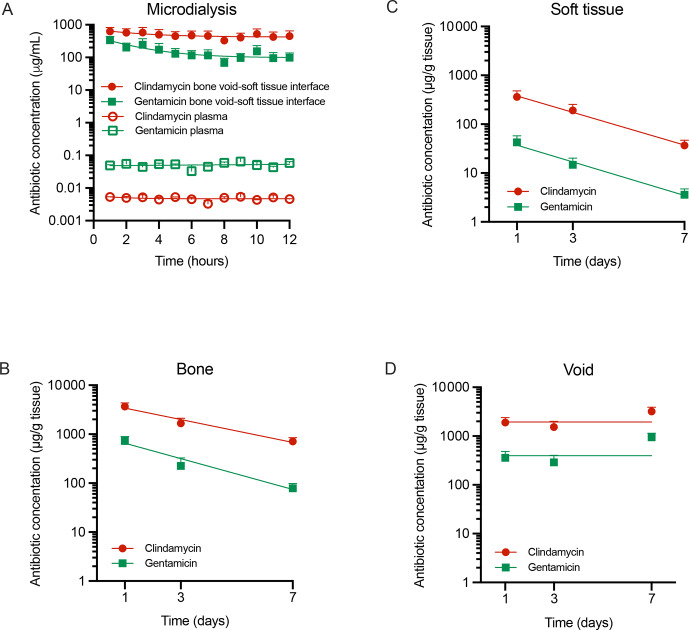
Antibiotic tissue concentrations. Concentrations of free clindamycin and gentamicin in **(A)** plasma and at the bone void–soft tissue interface and total concentrations in samples of **(B)** bone tissue, **(C)** soft tissue, and **(D)** bone void hematoma over 1–7 d. Data are presented as means ± SEM. Microdialysis bone–soft tissue interface: n= 4, microdialysis plasma: n= 2, soft tissue: n= 9–12 (3 values < LOQ for gentamicin day 7), bone tissue: n= 14–15 (1 value < LOQ for gentamicin day 7), and void hematoma: n= 7–9. LOQ: limit of quantification.

Microdialysis at the bone void–soft tissue interface revealed high initial free antibiotic concentrations, peaking at 340 ± 100 µg mL^−1^ for gentamicin and 620 ± 190 µg mL^−1^ for clindamycin, followed by a gradual decline to ∼ 100 and 450 µg mL^−1^, respectively (Fig. 2A). From day 1 to day 7, total tissue concentrations remained elevated. Gentamicin and clindamycin reached maximum levels on day 1 in bone (730 ± 170 and 3700 ± 700 µg g^−1^) and soft tissue (42 ± 15 and 360 ± 120 µg g^−1^) and on day 3 in void hematoma (940 ± 200 and 3200 ± 700 µg g^−1^) (Table 1). Bone and soft tissue exhibited half-lives of 42–46 h for gentamicin and 43–62 h for clindamycin. By day 7, concentrations remained above 79 ± 19 µg g^−1^ gentamicin and 710 ± 140 µg g^−1^ clindamycin in bone (Fig. 2B) and 3.6 ± 1.2 and 37 ± 10 µg g^−1^ in soft tissue (Fig. 2C). Bone void concentrations were stable throughout, exceeding 290 ± 110 µg g^−1^ gentamicin and 1500 ± 500 µg g^−1^ clindamycin (Fig. 2D). Clindamycin, administered at a 3.8-fold higher dose, yielded 3.2-fold greater free drug exposure (AUC_1–12 h_ ratio) and 4.1-, 6.5-, and 10.9-fold higher total exposure in bone void, bone, and soft tissue, respectively (AUC_1–7 d_ ratios).

**Table 1:** AUC, $C_{\max}$ , and $T_{1/2}$ .

	Clindamycin			Gentamicin		
	AUC	$C_{\max}$	$T_{1/2}$	AUC	$C_{\max}$	$T_{1/2}$
Free concentrations						
Plasma (1–12 h)	0.053 ± 0.003	0.0054 ± 0.0014	–	0.56 ± 0.04	0.065 ± 0.020	–
Bone-soft tissue interface (1–12 h)	5200 ± 900	620 ± 190	–	1610 ± 330	340 ± 100	–
Total concentrations						
Soft tissue (1–7 d)	24 000 ± 15 000	360 ± 120	43	2200 ± 1600	42 ± 15	42
Bone tissue (1–7 d)	240 000 ± 110 000	3700 ± 700	62	37 000 ± 26 000	730 ± 170	46
Bone void (1–7 d)	310 000 ± 130 000	3200 ± 700	–	75 000 ± 34 000	940 ± 200	–

AUC_(1–12 h)_ ( µg mL ^−1^ h), AUC_(1–7 d)_ ( µg g^−1^ h), $C_{\max}$ (1–12 h) ( µg mL^−1^), $C_{\max}$ (1–7 d) ( µg g^−1^), $T_{1/2}$ (h), Data: mean ± SEM.

### 3.2 Systemic antibiotic measurements

Plasma concentrations remained low and stable during the first 12 h (gentamicin: 0.033–0.065 µg mL^−1^; clindamycin: 0.0032–0.0054 µg mL^−1^) (Fig. 2A). Despite a 3.8-fold higher dose, clindamycin exposure was 10-fold lower than gentamicin (AUC_1–12 h_, Table 1). Beyond 24 h, plasma levels were below the LOQ. The highest tissue concentrations were detected in the kidney (gentamicin: 3.0 ± 1.9 µg g^−1^; clindamycin: 1.2 ± 0.7 µg g^−1^), while clindamycin was the only drug quantifiable in muscle (5 ± 4 µg g^−1^) and liver (0.5 ± 0.1 µg g^−1^) (Table A1).

### 3.3 Antibiotic release from depots

**Figure 3:**
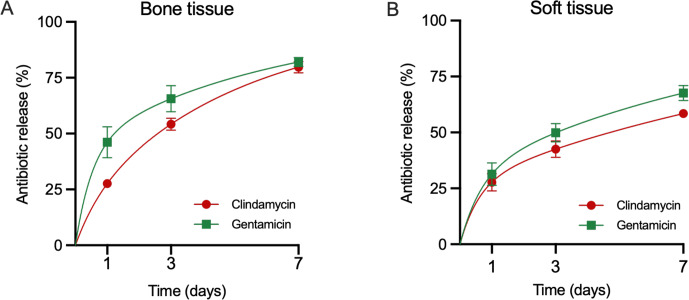
Antibiotic release from depots. Release of gentamicin and clindamycin from CarboCell G/C depots in bone **(A)** and soft tissue **(B)** at 1, 3, and 7 d in pigs. Mean ± SEM, bone: n= 3–5, soft tissue: 4–6.

**Figure A1:**
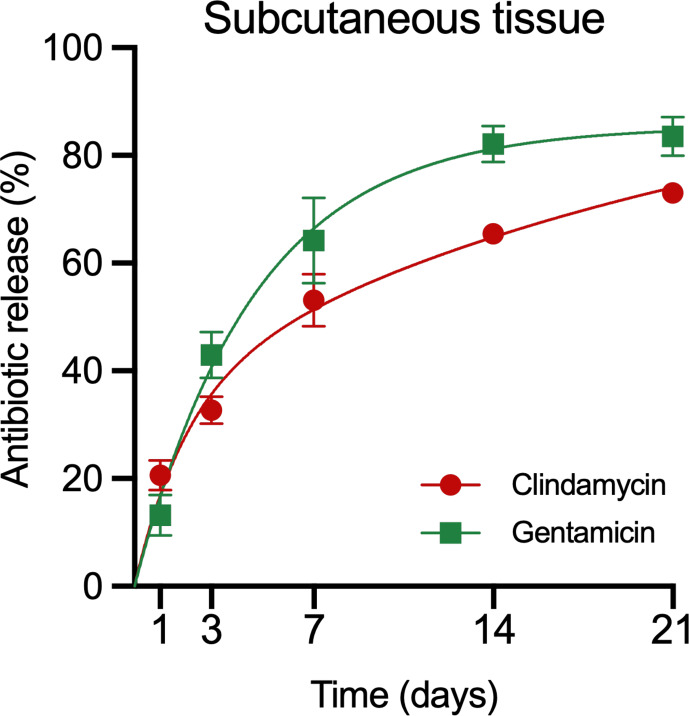
Antibiotic release from depots in mice. Accumulated release of gentamicin and clindamycin from CarboCell G/C depots in subcutaneous tissue at 1, 3, 7, 14, and 21 d after administration in mice. Data are presented as means and SEM. n= 5–6 depots at each timepoint (Balb/C mice, female, 8 weeks old (Janvier labs France), acclimatized at least 7 d before commencing study procedures, ad lib access to water and food, housed and monitored according to guidelines from the Danish Animal Inspectorate).

Antibiotic release from CarboCell G/C in pigs demonstrated an initial phase of faster release on day 1, with 46 ± 7 % of gentamicin and 28 ± 2 % of clindamycin released in bone and 31 ± 5 % of gentamicin and 28 ± 4 % of clindamycin released in soft tissue, followed by a slower release over the remaining study period (Fig. 3). By day 7, cumulative release in bone reached 82 ± 2 % for gentamicin and 80 ± 2 % for clindamycin (Fig. 3A), while in soft tissue, release was slightly lower at 68 ± 3 % for gentamicin and 58 ± 1 % for clindamycin (Fig. 3B). Continued release beyond day 7 was confirmed in mice. These data should be interpreted solely as qualitative support rather than as a directly comparable pharmacokinetic model (Fig. A1).

### 3.4 Morphological bone response

**Figure 4:**
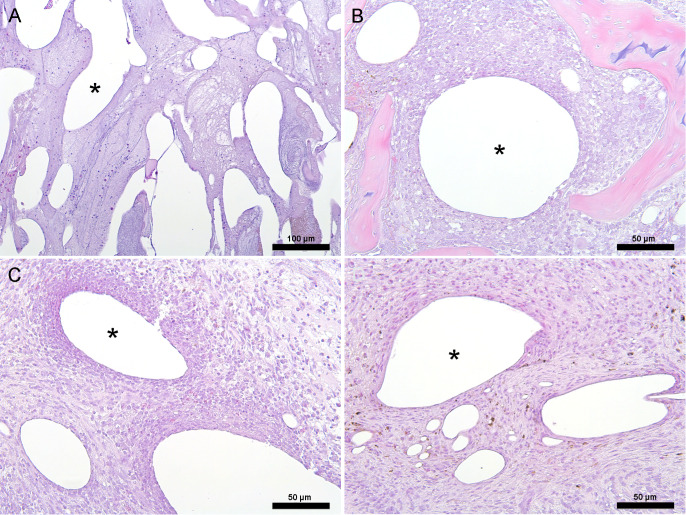
Histology. Representative images from pigs euthanized at days 1, 3, and 7, depicting the histological tissue response to CarboCell G/C depots. **(A)** Day 1: inflammatory response with mononuclear cells and neutrophils throughout the bone void, **(B)** day 3: mild localized response with fibroblasts and mononuclear cells in bone adjacent to the bone void, **(C–D)** day 7: response varying from a mild localized response **(D)** to a heavier localized mononuclear and neutrophilic inflammatory response **(C)** with more fibroplasia in bone adjacent to the bone void. Images **(C)** and **(D)** are from the same animal. ^*^ Examples of CarboCell G/C depots. Scale bars correspond to 100 µm in **(A)** and 50 µm in **(B)**–**(D)**.

CarboCell G/C depots were present within the bone void and surrounding tissue. Within the void, CarboCell G/C was intermingled with varying amounts of bone debris, fibrin, red blood cells, mononuclear cells, and neutrophils (Fig. 4A). The tissue response was not evaluated on day 1 due to the difficulty in reliably distinguishing bone marrow cells from an early inflammatory response. On day 3, the reaction to CarboCell G/C was mild, consisting of mononuclear cells, such as macrophages, and fibroblasts (Fig. 4B). On day 7, the response varied, with some depots closest to the bone void having a moderate infiltration of mainly fibroblasts and mononuclear cells but also some neutrophils, while others were associated with a mild inflammatory response of similar cells (Fig. 4C–D). The fibrosis score (day 3: 0.47 ± 0.15, day 7: 3.3 ± 0.67), osteoclast number (day 3: 0.13 ± 0.15, day 7: 1.6 ± 1.4), and presence of osteoid increased over time.

## 4 Discussion and conclusion

This study demonstrates that local delivery of gentamicin and clindamycin using CarboCell G/C maintains high antibiotic levels in the targeted bone void for at least 7 d, and recovered depots released approximately ∼ 30 %–50 % of their antibiotic content within the first day, reaching ∼ 60 %–80 % cumulative release by day 7. Prior evidence shows that CarboCell G/C effectively eradicates infection in a porcine model of implant-associated osteomyelitis (Henriksen et al., 2024), where systemic antibiotics (Vittrup et al., 2023) and a commercially available gentamicin-eluting hydroxyapatite and calcium sulfate biomaterial (Blirup-Plum et al., 2020) have failed. The present study further confirms that CarboCell G/C delivers therapeutic levels of antibiotics to the infection site, with negligible antibiotic spillover to plasma and off-target tissues, e.g. gentamicin levels remaining below the nephrotoxic limit throughout (Selby et al., 2009). We also observed that CarboCell G/C depots elicited a mild–moderate inflammatory response in bone tissue. Depots with a more pronounced cellular response were located at the bone–void interface, likely reflecting the inflammatory response to surgery. These findings align with previous observations in infected tissue after CarboCell G/C treatment (Henriksen et al., 2024; Fuglsang-Madsen et al., 2024).

The pharmacokinetic data for CarboCell G/C demonstrate clear advantages and robustness over other antibiotic administration forms relevant to orthopedic infections. For example, intravenous gentamicin in pigs resulted in an AUC_0–6 h_ of 26 µg mL^−1^ h and $C_{\max}$ of 6.9 µg mL^−1^ in cancellous bone (Stolle, 2004). Despite the 5.7-fold lower dose, CarboCell G/C resulted in markedly higher bone exposure while maintaining low systemic levels, highlighting its efficiency for local and targeted delivery. Similarly, intraosseous administration of 500 mg diluted vancomycin in pigs exhibited a half-life of 4.6 h in cancellous bone, while CarboCell G/C yielded prolonged half-lives of 46–62 h and markedly higher exposure when quantified over 7 d (CarboCell G/C AUC_1–7 d_: 37 000 µg g^−1^ h, intraosseous AUC_0–12 h_: 6244 µg mL ⋅ h^−1^), despite providing a 3–12 times lower antibiotic payload (Olsen Kipp et al., 2021). Further, our findings build upon a recent study demonstrating that intraosseous injection of CarboCell G/C can achieve high cancellous bone concentrations over at least 12 h with minimal systemic spillover (Mikkelsen et al., 2025). The concentrations measured in target tissue exceeded typical MIC values for common orthopedic pathogens such as *S. aureus* by several orders of magnitude, supporting the likelihood of effective antimicrobial activity at the target site. Unfortunately, comparable in vivo pharmacokinetic data for other carrier systems, including PMMA and hydroxyapatite and calcium sulfate-based carriers, are currently lacking.

Notably, in this study, clindamycin showed a 10-fold lower systemic exposure than gentamicin, despite being administered at a 3.8-fold higher dose. This may partly be explained by clindamycin's higher protein binding, which limits the free, systemically available fraction (Lee et al., 2013). Overall, lower antibiotic concentrations were observed in soft tissue compared to the bone void and adjacent cancellous bone, likely due to the smaller CarboCell G/C volume administered. This may reflect a balance between ongoing antibiotic release from surrounding CarboCell G/C depots and local washout kinetics, potentially combined with fluid dynamics that preferentially transport antibiotics toward the void space (Schwarz et al., 2021). Higher antibiotic levels in soft tissue could potentially have been achieved by placing multiple CarboCell G/C depots across a broader area.

By combining in vivo release profiles with tissue concentration data, this study clarifies the relationship between drug release, tissue exposure, and diffusion characteristics. Many reports on antibiotic-eluting technologies claim sustained release based on simple in vitro assays, where depots are placed in buffer and time above the minimum inhibitory concentration (MIC) is measured. Such assays fail to reflect physiological washout kinetics. Reported durations above MIC may instead result from assay-specific factors like buffer volume, depot size, or media exchange timing.

We recognize that this study has several limitations. The antibiotic concentrations were assessed using two quantification methods and interpreted accordingly. Microdialysis samples the free, pharmacologically active fraction of a drug in the extracellular compartment (Azeredo et al., 2014), whereas tissue extraction quantifies total drug concentrations without distinguishing between intra- and extracellular compartments or free and protein-bound fractions (Mouton et al., 2007). The tissue extraction method was optimized by performing triple extractions to ensure accurate quantification of total antibiotic levels for clindamycin and gentamicin (Russo et al., 2023). Gentamicin's low protein binding compared to clindamycin likely affects distribution and detection across sampling methods. Microdialysis may better represent gentamicin's tissue exposure, while for clindamycin it reflects only a fraction of total levels. This aligns with previous findings, showing that gentamicin pharmacokinetics in dialysates closely match bone sample data (Stolle, 2004). Moreover, since the extracellular compartment accounts for 98 %–99 % of bone tissue volume, dilution from intracellular contents during homogenization is minimal, supporting the fact that measured bone concentrations closely approximate extracellular levels (Pea, 2009). Also, the antibiotic concentrations were measured in healthy tissue, which may differ from infected tissue (Zhao et al., 2019). Finally, given that CarboCell G/C is biodegradable, longer-term studies on antibiotic delivery and biocompatibility are warranted.

In conclusion, CarboCell G/C achieved high, sustained local concentrations of clindamycin and gentamicin for at least 7 d with minimal systemic exposure. This technology may improve treatment efficacy while reducing reliance on systemic antibiotics and the risk of antimicrobial resistance.

## Data Availability

Data are available from the corresponding author.

## Appendix A

**Table A1:** Systemic total antibiotic concentrations.

	Clindamycin ( n= 3)			Gentamicin ( n= 3)		
	Day 1	Day 3	Day 7	Day 1	Day 3	Day 7
Muscle ( µg g^−1^)	5 ± 4^*^	< LOQ	< LOQ	< LOQ	< LOQ	< LOQ
Kidney ( µg g^−1^)	NR	NR	1.2 ± 0.7^*^	3.0 ± 1.9^*^	2.1 ± 0.8	2.3 ± 0.9
Liver ( µg g^−1^)	NR	< LOQ	0.5 ± 0.1^*^	< LOQ	< LOQ	< LOQ
Plasma surgery ( µg mL^−1^)	< LOQ	< LOQ	< LOQ	< LOQ	< LOQ	< LOQ
Plasma euthanasia ( µg mL^−1^)	< LOQ	< LOQ	< LOQ	< LOQ	< LOQ	< LOQ

LOQ: limit of quantification, NR: not reported due to only 1 value above LOQ. ^*^ n= 2. Data are presented as mean ± SEM.
